# Association of peripheral inflammatory cytokines with motor and non-motor symptoms in patients with Parkinson’s disease and type 2 diabetes mellitus

**DOI:** 10.3389/fneur.2025.1474812

**Published:** 2025-07-02

**Authors:** Aiping Hu, Yuqing She, Xue Cao, Yang Wang, Shu Wu, Juan Lu, Yang Zhao, Lizhi Yu, Haifeng Jiang, Qing Chen

**Affiliations:** ^1^Department of Endocrinology, Nanjing Pukou People’s Hospital, Liangjiang Hospital Southeast University, Nanjing, Jiangsu, China; ^2^Department of Encephalopathy, Nanjing Hospital of Chinese Medicine Affiliated to Nanjing University of Chinese Medicine, Nanjing, Jiangsu, China; ^3^Department of Neurology, Nanjing Pukou People’s Hospital, Liangjiang Hospital Southeast University, Nanjing, Jiangsu, China; ^4^Department of Encephalopathy, Nanjing Pukou District Hospital of Chinese Medicine, Nanjing, Jiangsu, China

**Keywords:** Parkinson’s disease, type 2 diabetes mellitus, inflammatory cytokines, motor symptoms, non-motor symptoms

## Abstract

**Objective:**

This pilot study aims to investigate the association between peripheral inflammatory cytokines and motor and non-motor symptoms in patients with both Parkinson’s disease (PD) and type 2 diabetes mellitus (T2DM) and the underlying mechanisms.

**Methods:**

Sixty patients with PD were divided into two groups depending on whether they also had T2DM, resulting in a PD group (21 cases) and a PD–T2DM group (39 cases). Thirty healthy volunteers from the physical examination centre were enrolled as the control group. Peripheral blood was collected from all patients.

**Results:**

Patients with PD–T2DM had higher Movement Disorders Society Unified Parkinson’s Disease Rating Scale (MDS-UPDRS) III scores; total MDS-UPDRS scores; Parkinson’s Disease Questionnaire-39 (PDQ-39) scores; and interleukin (IL)-6, IL-1β, tumour necrosis factor alpha (TNF-*α*) and IL-4 levels than patients with PD (*p* < 0.05). In the PD group, IL-4 levels correlated with UPDRS II (*r* = 0.337), Non-Motor Symptom Scale (*r* = 0.354), Hamilton Depression Scale (*r* = 0.420) and PDQ-39 (*r* = 0.423) scores (*p* < 0.05). A multivariate regression revealed IL-6 independently predicted lower UPDRS III scores (*β* = −0.497, *p* = 0.018), TNF-*α* correlated with PD duration (*β* = 0.689, *p* < 0.001) and IL-1β correlated with PDQ-39 scores (*β* = 0.462, *p* = 0.002) in patients with PD–T2DM. Adjusted models explained up to 52.3% of variance (adjusted R^2^). In the PD group, age-adjusted correlations confirmed IL-4 was associated with UPDRS II (*r* = 0.321, *p* = 0.047) and PDQ-39 (*r* = 0.418, *p* = 0.009), and interferon gamma (IFN-*γ*) was associated with Scales for Outcomes in Parkinson’s Disease-Autonomic Questionnaire (SCOPA-AUT; *r* = −0.564, *p* = 0.001). Negative correlations were identified between IL-6 and UPDRS III scores (*r* = −0.497) and IFN-*γ* and SCOPA-AUT scores (*r* = −0.588; *p* < 0.05).

**Conclusion:**

These pilot findings suggest peripheral inflammatory cytokines can be considered biomarkers in patients with PD–T2DM. The underlying mechanism by which T2DM worsens the motor and non-motor symptoms of PD may involve increased inflammation.

## Introduction

Parkinson’s disease (PD) is a chronic, gradually progressive and neurodegenerative disorder primarily characterised by decreased motor activity due to a loss of dopaminergic neurons in the substantia nigra. In addition to the classic motor symptoms of resting tremor, bradykinesia and myotonia, patients with PD experience several non-motor symptoms, including autonomic dysfunction and sleep, sensory, psychiatric and cognitive disturbances. The development of tools such as the Non-Motor Symptom Scale (NMSS) allows physicians to quantify the overall burden of different symptoms on patients with PD and their impact on patients’ quality of life, which can be helpful in the early identification and treatment of PD. Parkinson’s disease affects approximately 1% of adults aged ≥65 years. This increases to 4–5% in adults aged >85 years (1). Type 2 diabetes mellitus (T2DM) is a chronic metabolic disorder in which the pancreas is unable to produce adequate insulin or the body resists insulin. Although age is an important risk factor for PD, evidence shows that T2DM is also associated with an increased PD risk and may contribute to the faster progression of motor and non-motor PD symptoms (2). However, the molecular mechanisms underlying the interplay between the pathogenesis of T2DM and PD remain unclear. Common potential mechanisms between them may include insulin resistance, inflammation, mitochondrial dysfunction, endoplasmic reticulum stress, autophagy and the ubiquitin–proteasome system (3).

Inflammation is a highly regulated process that prevents tissue damage and is associated with the pathogenesis of T2DM and PD (4). Neuroinflammation plays an important role in PD pathogenesis and development. Activated microglia and astrocytes worsen neuroinflammation and are critical in PD progression (3). Inflammatory cytokines include pro-inflammatory cytokines, such as interleukin (IL)-1β, IL-6, tumour necrosis factor alpha (TNF-*α*) and interferon gamma (INF-*γ*), and anti-inflammatory cytokines, such as IL-4 and IL-10. A case–control study of 58 patients with PD and 20 healthy controls revealed that IL-1β, IL-2 and IL-6 were significantly elevated in patients with PD compared with the controls (5). Moreover, Chen et al. (6) indicated that patients with PD had increased levels of transforming growth factor beta 1, IL-1β and IL-6 in their cerebrospinal fluid. Malik et al. (7) reported that, compared with controls, patients with T2DM had elevated levels of TNF-*α*, IL-6 and anti-inflammatory cytokine IL-10. These studies suggest that inflammation may play a vital role in PD and T2DM. Previous studies have implicated inflammation in PD symptomatology (8–10), including motor symptoms, depression and quality of life. However, to the best of our knowledge, no studies have focused on the expression of inflammatory factors and the relationship between inflammation and motor and non-motor symptoms in patients with both PD and T2DM (PD–T2DM).

To address these knowledge and literature gaps, we conducted this exploratory cross-sectional pilot study to characterise serum inflammatory cytokines and explore whether these cytokines are associated with motor and non-motor symptoms in patients with PD and in those with PD–T2DM.

## Methods

### Participants

This was a cross-sectional study in which patients with PD were selected from the outpatient and inpatient departments of the Nanjing University of Chinese Medicine’s Affiliated Hospital, Nanjing Brain Hospital, Nanjing Pukou People’s Hospital and the Nanjing Pukou District Hospital of Traditional Chinese Medicine between June 2022 and June 2023. Ninety individuals were included in the study and divided into three groups: a control group (30 healthy individuals), a PD group (39 patients with PD) and a PD–T2DM group (21 patients with both PD and T2DM). An *a priori* power calculation was not performed due to the nature of the study design, which was based on the available cases at the time of the study. The aim was to capture a representative snapshot of the patient population; thus, sample size was determined by the number of eligible patients identified within the study period.

The inclusion criteria were as follows: (1) individuals aged 50–90 years; (2) a clinical diagnosis of PD based on the United Kingdom Parkinson’s Disease Society Brain Bank criteria, which include delayed movement and at least one of the following symptoms: muscle rigidity, resting tremor (4–6 Hz) or postural instability (not caused by primary visual, cerebellar, vestibular or proprioceptive dysfunction); (3) a diagnosis of T2DM based on the 2019 American Diabetes Association criteria (11) (a fasting plasma glucose [FPG] ≥ 126 mg/dL [7.0 mmol/L] or a 2-h plasma glucose ≥200 mg/dL [11.1 mmol/L] during an oral glucose tolerance test); (4) voluntary participation in this study and signed informed consent; and (5) no contraindications for treatment. Patients with PD–T2DM were required to have had T2DM prior to developing PD. The exclusion criteria were as follows: (1) patients with Parkinson–Plus syndrome; (2) patients with gestational diabetes, type 1 diabetes or special-type diabetes; (3) serious complications, chronic inflammatory diseases (such as rheumatoid arthritis) or a mental illness that made it difficult to complete clinical trials; and (4) pregnant or lactating women. Given the exploratory nature of this pilot investigation aimed at initial characterisation, an *a priori* power calculation was not performed. The sample size was determined by the number of eligible patients identified within the study period at the participating centres. We acknowledge this limits statistical power and generalizability. Consequently, this study should be interpreted as a hypothesis-generating pilot investigation.

Data collected included sex; age; body mass index; glycosylated haemoglobin; fasting blood glucose; serum islet; IL-1β, IL-4, IL-6, IL-10, IFN-*γ* and TNF-*α* levels; PD duration; and FPG. The study protocol was approved by the Ethics Committee of Nanjing Pukou People’s Hospital (Approval Number: 2023-SR − 008). This study was conducted in accordance with the principles of the Declaration of Helsinki. All the participants provided written informed consent.

### Measurement of inflammatory cytokines

After patients had fasted for 10–12 h, peripheral venous blood samples of 5 mL were taken. After centrifugation at 3,000 r/min for 15 min, the upper serum was collected and put into clean Eppendorf tubes. The serum was then frozen in the refrigerator at −80°C for later detection. The clinical data of patients corresponding to the serum samples were also recorded for later statistical analysis. Enzyme-linked immunosorbent assay (ELISA) was used to detect the levels of IL-1β, IL-4, IL-6, IL-10, IFN-*γ* and TNF-*α* in human serum. The human serum samples and ELISA kit (Hunan Aifang Biotechnology Co., Ltd., Changsha, China) were left at room temperature for 1 h to thaw in advance. The solution was added, and the samples were placed in a Multiskan SkyHigh™ photometer (Thermo Scientific, A51119500C) within 5 min. Wavelength was set to 450 nm, absorbance was detected and the corresponding concentrations of each indicator in the sample were calculated based on a calibration curve.

### Assessment of motor and non-motor symptoms

Motor symptoms were evaluated using the Chinese version of the Movement Disorders Society Unified Parkinson’s Disease Rating Scale (MDS-UPDRS) II and III, total MDS-UPDRS and the Hoehn and Yahr scale (H&Y) (12). Non-motor symptoms were evaluated using the Chinese version of the NMSS (13), the Hamilton Depression Scale (HAMD) (14), the Parkinson’s Disease Sleep Scale (PDSS) (15), the Scales for Outcomes in Parkinson’s Disease-Autonomic Questionnaire (SCOPA-AUT) (16) and the Parkinson’s Disease Questionnaire-39 (PDQ-39) (17). The Chinese version of the Parkinson’s Disease Pattern Element Scale-13 (PD-PES-13) (18) was used to assess the characteristics of the TCM syndrome elements of PD.

### Quality control

To control all kinds of biases effectively, strict inclusion and exclusion criteria were formulated. All research participants in the case group were repeatedly confirmed by at least two experienced senior professional title experts who had been systematically trained. The recognised UPDRS scoring form, H&Y staging standard and unified basic information questionnaire were adopted to accurately record the basic information of each research patient in detail to ensure the reliability of each item of information. At least two staff members input and corrected data simultaneously in the data collection stage and checked existing data regularly to minimise the influence of various biases.

### Statistical analysis

Data were analysed using IBM SPSS software version 22.0. Normality was assessed via the Shapiro–Wilk test and Q–Q plots. Normally distributed continuous variables were expressed as mean ± standard deviation, whereas skewed variables were reported as medians (25^th^–75^th^ percentiles). Categorical variables were summarised as frequencies (%). Group comparisons utilised independent t-tests, ANOVA (with Bonferroni correction for pairwise comparisons), Mann–Whitney U tests or Kruskal–Wallis tests, as appropriate. Chi-squared tests were applied for categorical data. Partial correlations between inflammatory cytokines and clinical measures were calculated, adjusting for age and sex, and sensitivity analyses for the PD group further evaluated age-only adjustments. To address confounding effects, multiple linear regression models were constructed for the PD–T2DM group, incorporating inflammatory cytokines (IL-6, IL-1β, TNF-*α*, IL-4, IL-10 and IFN-*γ*) as predictors and adjusting for age, sex, PD duration and FPG. Multicollinearity was verified using variance inflation factors (<5), and residual diagnostics confirmed model assumptions. All tests were two-tailed, with *p* < 0.05 deemed statistically significant.

## Results

A total of 39 patients with PD, 21 patients with PD–T2DM and 30 healthy controls were included in this study. Patient demographics and clinical characteristics are summarised in Table 1. There was no difference between the three groups with respect to sex (*p* = 0.101). Significant differences were observed between the three groups with respect to FPG (*p* = 0.000) and age (*p* = 0.001). The PD–T2DM group had higher MDS-UPDRS III, total MDS-UPDRS and PDQ-39 scores than the PD group (*p* = 0.038, 0.029 and 0.044, respectively). There were no differences in PD duration (*p* = 0.689), H&Y stage (*p* = 0.933), MDS-UPDRS II score (*p* = 0.475), PDSS score (*p* = 0.846), SCOPA-AUT score (*p* = 0.192), PD-PES-13 score (*p* = 0.255), NMSS score (*p* = 0.299) or HAMD score (*p* = 0.235) between the PD and PD–T2DM groups.

**Table 1:** Participant demographics and clinical characteristics.

Variables	PD	PD-T2DM	Control	*p*
	(*N* = 39)	(*N* = 21)	(*N* = 30)	
Gender(male/female)	19 (48.7%)/20 (51.3%)	9 (42.9%)/12 (57.1%)	21 (70.0%)/9 (30.0%)	0.101
FPG(mg/dl)	86.26 ± 10.69	118.02 ± 37.36	96.40 ± 7.36	0.000***
Age(years)	69.00 (61.00, 72.00)	70.00 (66.50, 77.00)	61.00 (60.00, 67.25)	0.001**
PD duration (years)	7.85 ± 3.91	7.43 ± 3.70	NA	0.689
UPDRSIII score	38.38 ± 13.16	47.48 ± 19.95	NA	0.038*
PDSS score	16.82 ± 9.52	17.29 ± 7.27	NA	0.846
SCOPA-AUT score	14.82 ± 8.14	17.81 ± 8.79	NA	0.192
PD-PES-13 score	51.10 ± 19.02	57.69 ± 24.77	NA	0.255
H&Y stage	2.50 (2.00, 3.00)	3.00 (2.00, 3.00)	NA	0.933
UPDRSII score	13.00 (9.00, 19.00)	16.00 (10.50, 19.00)	NA	0.475
Total UPDRS score	64.00 (53.00, 81.00)	79.00 (66.50, 96.00)	NA	0.029*
NMSS score	44.00 (32.00, 94.00)	60.00 (37.00, 97.00)	NA	0.299
HAMD score	9.00 (5.00, 14.00)	11.00 (6.00, 15.00)	NA	0.235
PDQ39 score	29.00 (17.00, 48.00)	45.00 (25.00, 68.00)	NA	0.044*

Data presented as number, mean ± standard deviation, or median (range). FPG, fasting plasma glucose; MDS-UPDRS, Movement Disorder Society Unified Parkinson’s Disease Rating Scale; NMSS, Non-motor Symptoms Scale; HAMD, Hamilton Depression Scale; PDSS, Parkinson’s Disease Sleep Scale; SCOPA-AUT, Scales for Outcomes in Parkinson’s disease Autonomic; PDQ-39, Parkinson’s disease Questionnaire-39; PD-PES-13, Parkinson’s Disease Pattern Element Scale-13; H & Y, Hoehn and Yahr; PD, Parkinson’s disease; PD-T2DM, Parkinson’s disease with type 2 diabetes mellitus; TNF, tumour necrosis factor; IL, interleukin; IFN, interferon; NA, Not applicable. *p < 0.05, **p < 0.01, ***p < 0.001.

**Figure 1:**
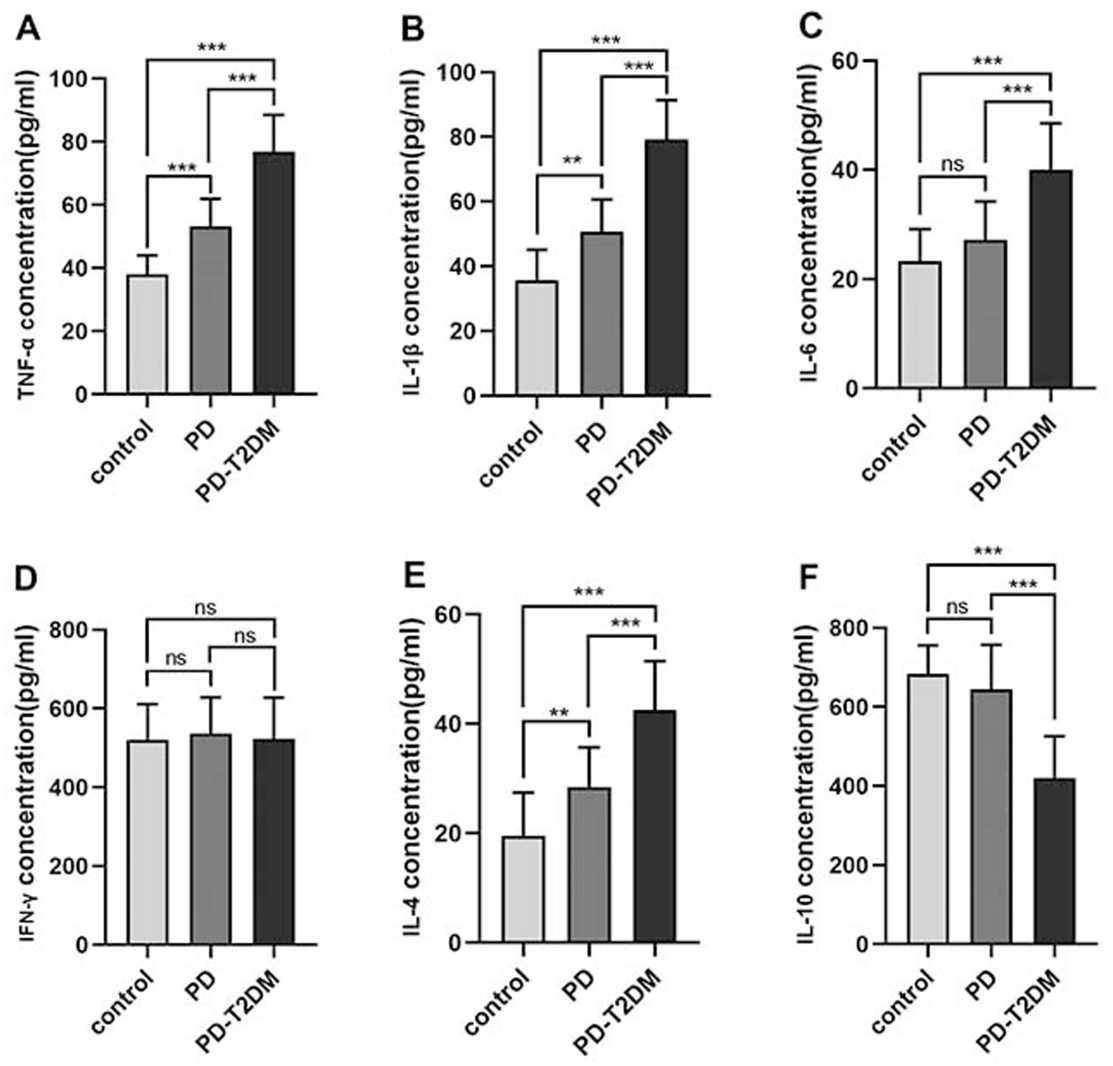
Levels of inflammatory cytokines in the PD, PD-T2DM, and control groups. Middle lines represent mean values, and T bars indicate standard deviation; **p < 0.01, ***p < 0.001, ns: no significant difference.

Inflammatory cytokine concentrations in all groups are shown in Figure 1. Levels of TNF-*α*, IL-6, IL-1β and IL-4 were highest in the PD–T2DM group and lowest in the control group. Pairwise comparisons among the three groups revealed significant differences; however, IL-10 expression showed the opposite trend. Surprisingly, no difference was noted in IFN-*γ* levels between the three groups.

To address confounding factors, multiple linear regression analyses were conducted in the PD–T2DM group (Table 2). After adjusting for age, sex, PD duration and FPG, UPDRS III scores were found to be independently associated with IL-6 (*β* = −0.497, 95% CI: −0.892 to −0.102, *p* = 0.018) and TNF-*α* (*β* = 0.689, 95% CI: 0.532–0.846, *p* < 0.001), with the model explaining 52.3% of variance (adjusted R^2^ = 0.523). Parkinson’s Disease Questionnaire-39 scores were significantly predicted by IL-1*β* (*β* = 0.462, 95% CI: 0.201–0.723, *p* = 0.002) and FPG (*β* = 0.312, *p* = 0.041), accounting for 38.7% of variance. Hamilton Depression Scale scores showed a positive correlation with IL-10 (*β* = 0.512, 95% CI: 0.128–0.896, *p* = 0.012), whereas SCOPA-AUT scores were inversely linked to IFN-*γ* (*β* = −0.588, 95% CI: −0.962 to −0.214, *p* = 0.003), with adjusted R^2^ values of 41.2 and 49.5%, respectively.

**Table 2:** Multiple linear regression analysis of inflammatory cytokines and clinical scores in PD-T2DM patients.

Dependent variable	Independent variable	β coefficient	95% CI	*p*-value	Adjusted R^2^
UPDRS III	IL-6	−0.497	−0.892 to −0.102	0.018*	0.523
	TNF-α	0.689	0.532 to 0.846	<0.001***	
PDQ-39	IL-1β	0.462	0.201 to 0.723	0.002**	0.387
	FPG	0.312	0.014 to 0.610	0.041*	
HAMD	IL-10	0.512	0.128 to 0.896	0.012*	0.412
SCOPA-AUT	IFN-γ	−0.588	−0.962 to −0.214	0.003**	0.495

*All models adjusted for age, sex, PD duration, and FPG; *p < 0.05, **p < 0.01, ***p < 0.001.

**Figure 2:**
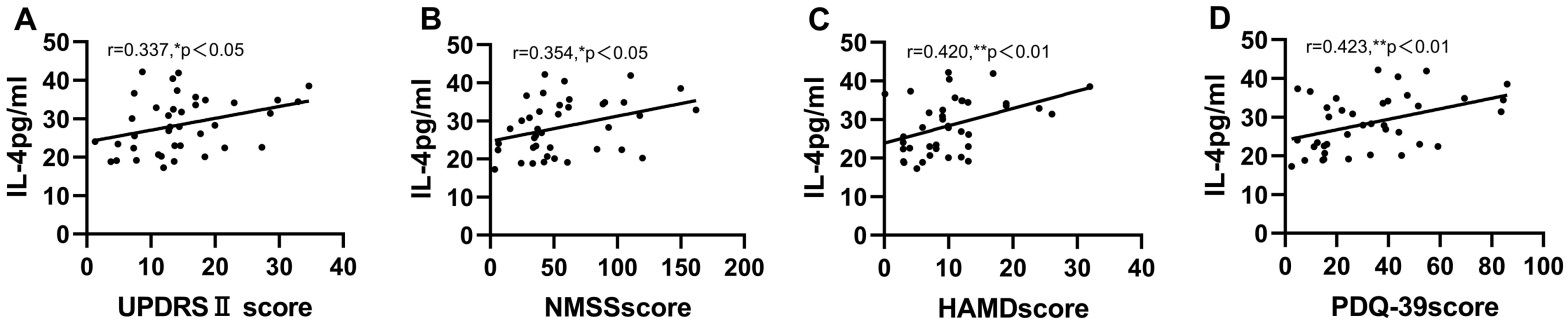
Serum inflammatory cytokine IL-4 correlates with motor and non-motor scores in PD patients. IL-4 was positively correlated with UPDRSII score, NMSS score, HAMD score, PDQ-39 score **(A–D)**. *p < 0.05, **p < 0.01. Partial correlation, corrected for gender and age.

The partial correlation between clinical variables and serum inflammatory cytokine levels is shown in Figure 2. Concerning motor and non-motor symptoms in patients with PD, IL-4 was positively correlated with UPDRS II (*r* = 0.337, *p* < 0.05), NMSS (*r* = 0.354, *p* < 0.05), HAMD (*r* = 0.420, *p* < 0.01) and PDQ-39 (*r* = 0.423, *p* < 0.01) scores after correcting for sex and age, unless otherwise specified. However, no correlation was found between the levels of other inflammatory cytokines and variables.

To enhance the robustness of correlation findings in the PD group, partial correlations were re-analysed by adjusting for age alone, given the non-significant sex distribution differences between groups (*p* = 0.101; Table 3). Interleukin-4 remained positively correlated with UPDRS II (*r* = 0.321, *p* = 0.047), NMSS (*r* = 0.338, *p* = 0.036), HAMD (*r* = 0.407, *p* = 0.011) and PDQ-39 (*r* = 0.418, *p* = 0.009) scores. These associations were consistent with the original analysis adjusting for both sex and age. Interleukin-6 showed a weaker negative correlation with UPDRS III (*r* = −0.452, *p* = 0.005, vs. the original *r* = −0.497). Interferon gamma maintained a significant negative correlation with SCOPA-AUT scores (*r* = −0.564, *p* = 0.001). Notably, adjusting for age alone did not substantially alter the direction or significance of correlations when compared with adjustments for both sex and age, suggesting the minimal confounding effect of sex in this cohort.

**Table 3:** Partial correlation analysis in the PD group adjusted for age.

Cytokine	Clinical Measure	Partial r	*p*-value
IL-4	UPDRS II	0.321	0.047*
	NMSS	0.338	0.036*
	HAMD	0.407	0.011*
	PDQ-39	0.418	0.009**
IL-6	UPDRS III	−0.452	0.005**
IFN-γ	SCOPA-AUT	−0.564	0.001***

*Partial correlations adjusted for age; *p < 0.05, **p < 0.01, ***p < 0.001.

**Figure 3:**
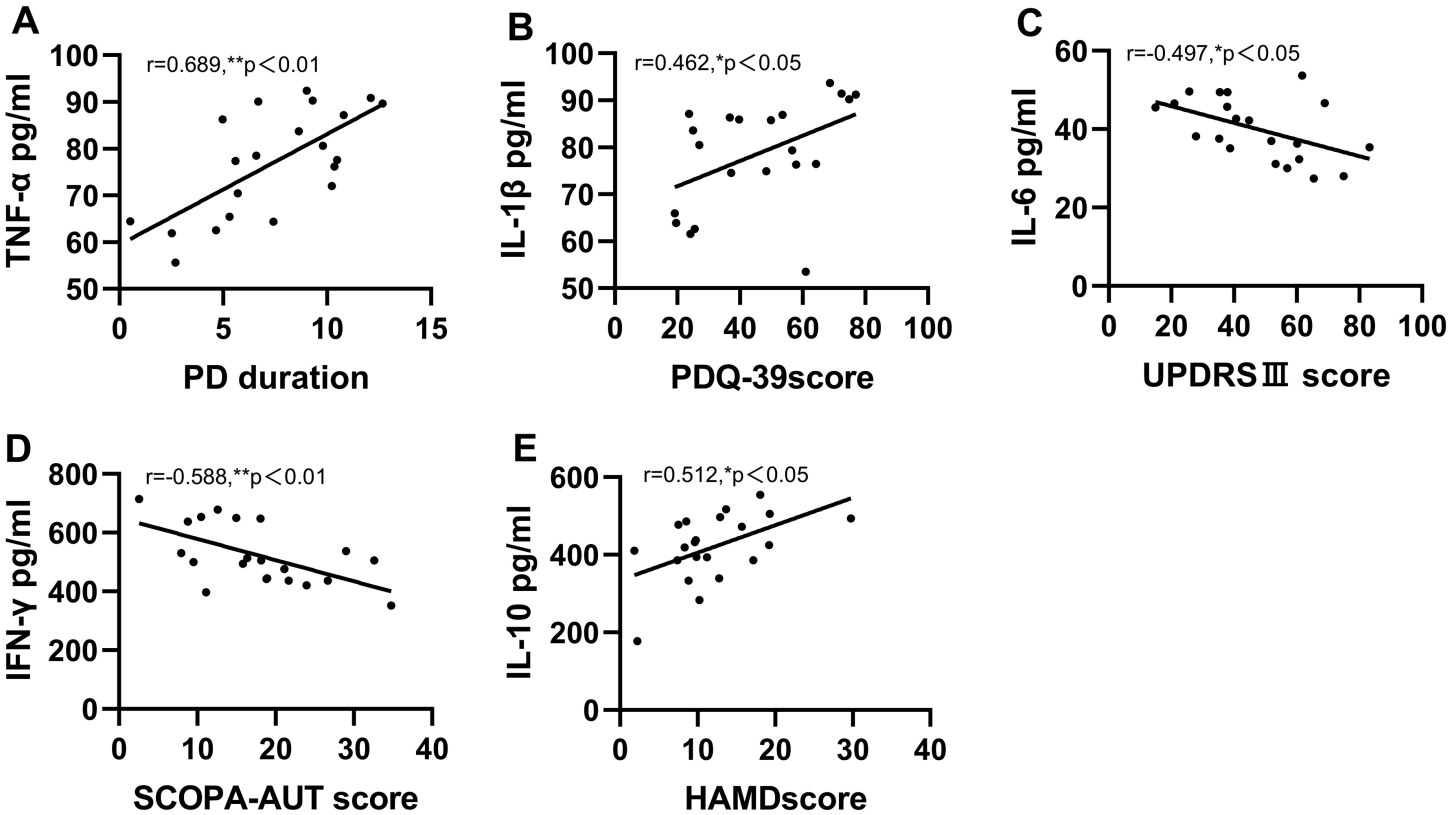
Serum inflammatory cytokines correlate with motor and non-motor scores in PD-T2DM patients. **(A)** TNF-*α* level positively correlates with PD duration. **(B)** IL-1*β*level positively correlates with PDQ-39 score. **(C)** Serum IL-6 level negatively correlates with UPDRSIII score. **(D)** IFN-*γ* level negatively correlates with SCOPA-AUT score. **(E)** Serum IL-10 level positively correlates with HAMD score. *p < 0.05, **p < 0.01. Partial correlation, corrected for gender and age.

The partial correlation analyses of patients with PD–T2DM are presented in Figure 3. Correlations were corrected for age and sex, unless otherwise specified. Positive correlations were found between TNF-*α* and PD duration (*r* = 0.689, *p* < 0.01), IL-1β and PDQ-39 score (*r* = 0.462, *p* < 0.05) and IL-10 and HAMD score (*r* = 0.512, *p* < 0.05); however, negative correlations were found between IL-6 and UPDRS III score (*r* = −0.497, *p* < 0.05) and IFN-*γ* and SCOPA-AUT score (*r* = −0.588, *p* < 0.01). The correlation between IL-4 and other variables showed no significant difference. There are no statistics for missing data.

## Discussion

Previous studies (2, 19) have found that T2DM can induce a more aggressive phenotype in PD, which is consistent with our findings. We found that patients with PD–T2DM had higher MDS-UPDRS and PDQ-39 scores than those with PD alone. However, whether the effects of T2DM on PD are additive or interactive remains uncertain. Pagano et al. (19) reported that, compared with patients with PD, patients with PD–T2DM exhibited higher motor scores, less striatal dopamine transporter binding and higher cerebrospinal fluid tau levels. Type 2 diabetes mellitus exacerbates the development of motor deficits and cognitive impairment in patients with PD. Wang et al. indicated that metabolic inflammation accelerated dopaminergic neuronal degeneration and increased the activation of nuclear factor kappa B and the nod-like receptor pyrin 3 inflammasome in the midbrain, aggravating neuroinflammation in 1-methyl-4-phenyl-1, 2, 3, 6-tetrahydropyridine (MPTP)-treated type 2 diabetic mice (20). These studies support the idea that inflammation may be a pathophysiological link between PD and T2DM, helping to elucidate why T2DM exacerbates the motor and non-motor symptoms of PD.

Numerous studies have confirmed that inflammatory cytokine levels are elevated in patients with PD (5, 6) and in those with T2DM (7), which is consistent with our findings. However, no studies have examined the expression of inflammatory factors in patients with both PD and T2DM. Wang’s animal study stands alone in demonstrating the increased expression of pro-inflammatory cytokines TNF-*α*, IL-6 and IL-1β and the decreased expression of anti-inflammatory cytokines IL-4 and IL-10 in the midbrain of MPTP-treated type 2 diabetic mice (21). In human studies, we initially revealed that changes in TNF-α, IL-6, IL-1β and IL-10 were consistent with Wang’s animal study, whereas the change in IL-4 exhibited an opposing pattern. Elevated serum TNF-α, IL-6 and IL-1β levels suggest increased inflammation in patients with PD–T2DM. The decrease in anti-inflammatory factor IL-10 suggests a diminished anti-inflammatory effect in patients with PD–T2DM. The contradictory result for IL-4 could be attributable to differences in specimens and the methods employed for measuring inflammatory factors. Peripheral blood inflammatory factors were measured using ELISA in human patients, whereas inflammatory cytokines were measured in the midbrain using quantitative real-time polymerase chain reaction in mice. Inflammation factors play a key role in the pathological mechanism of PD and T2DM (22, 23). They are involved in oxidative stress, neurodegeneration and insulin resistance, providing a theoretical basis for their use as biomarkers. This study indicates that levels of inflammatory factors may be related to the severity of motor and non-motor symptoms in PD, providing a potential indicator for monitoring the progress of the disease. At the early stage of the disease, a change in inflammatory factors may precede the appearance of clinical symptoms, providing the possibility of early diagnosis (24). A multiple regression analysis further confirmed that, after adjusting for confounding factors, specific inflammatory factors (such as IL-6, TNF-*α* and IL-1 *β*) were independently associated with motor and non-motor symptoms in patients with PD–T2DM. For example, the negative correlation between IL-6 and UPDRS III scores may reflect the anti-inflammatory compensatory mechanism of IL-6 in the central nervous system (CNS), whereas the positive correlation between TNF-*α* and disease progression suggests the cumulative effect of chronic inflammation on disease progression. In addition, the independent effect of IL-1β on quality of life (per PDQ-39 scores) supports the hypothesis that inflammation exacerbates symptoms through oxidative stress and insulin resistance.

Most studies have suggested a correlation between serum inflammatory cytokines and motor and non-motor symptoms in patients with PD. Kouchaki et al. (8) demonstrate that serum TNF-α levels were positively correlated with H&Y scales in patients with PD. A longitudinal study of 47 patients with PD confirmed that elevated IL-6 levels at baseline showed worse depression scores, and higher levels of C3 and C4 at baseline decreased quality of life after 2 years (9). Although Green et al. (25) reported negative results, increasing levels of serum inflammation cytokines may also be correlated with the motor and non-motor symptoms of PD. In our study, IL-4 was positively correlated with MDS-UPDRS II, NMSS, HAMD and PDQ-39 scores in patients with PD. Diaz et al. (10) revealed that IL-4 was positively correlated with motor symptom tremors in patients with PD. This is similar to our results. Other studies (9, 25) have also reported links between inflammatory factors and non-motor symptoms, such as depression and quality of life, in patients with PD. Interleukin-4 can exert a direct influence on beta cell function and viability as an anti-inflammatory molecule. In patients with diabetes, IL-4 levels have been shown to be higher than in patients in a control group (26). Interleukin-4, which in our study predicted motor and non-motor symptoms in patients with PD, may have dual functions in the CNS. Although IL-4 has been associated with the death of activated microglia and neuronal survival, it has also been shown to accelerate neurodegeneration in proinflammatory rats by promoting microglial activation and the production of IL-1β (10). By contrast, the lack of correlation between IL-4 and motor and non-motor symptoms may be due to its greater anti-inflammatory effect at higher levels in patients with PD–T2DM. A re-analysis of PD group correlations adjusted for age alone confirmed the stability of key associations (e.g., IL-4 with non-motor symptoms). The minimal impact of sex adjustment suggests that age is the dominant confounder in this cohort, likely due to its role in amplifying inflammatory pathways and disease progression. These findings underscore the importance of age as a critical covariate in studies of PD-related inflammation.

Although we did not demonstrate an association between inflammatory factors and motor and non-motor symptoms in patients with PD, our findings revealed positive correlations between TNF-*α* and PD duration, IL-1β and PDQ-39 score, and IL-10 and HAMD score and negative correlations between IL-6 and UPDRS III score and IFN-*γ* and SCOPA-AUT score in patients with PD–T2DM. Kouchaki et al. (8) revealed that TNF-α was positively correlated with PD duration in patients with PD. Our data demonstrate significantly higher IL-1β levels and PDQ-39 scores in the PD–T2DM group than in the PD group. Increased TNF-α and IL-1β levels in patients with PD–T2DM, which indicate aggravated inflammation, may explain the relationships between TNF-α, IL-1β, PD symptom duration and PDQ-39 scores in our study. We also found that IL-6 levels were negatively correlated with motor function in patients with PD–T2DM. However, Green et al. (25) identified a positive correlation between IL-6 and UPDRS III motor scores in patients with PD, which may be due to IL-6’s implication as a multifunctional cytokine in the pathophysiology of PD and T2DM.

Increased circulating levels of IL-6 may play a proinflammatory role, leading to the progression of PD pathophysiology, or an anti-inflammatory role, providing protection against other proinflammatory mechanisms (10). In patients with T2DM, the acute elevation of plasma IL-6 in circulation plays a protective role against systemic inflammation by promoting plasma levels of anti-inflammatory IL-10 and IL-1 receptor antagonist (27). In addition, our data suggest that lower levels of IFN-*γ* predict more severe SCOPA-AUT scores (i.e., non-motor symptoms) in patients with PD–T2DM. Diaz et al. (10) found that IFN-γ was negatively correlated with tremors (a motor symptom) in patients with PD. Similarly to IL-4, IFN-*γ* performs dual functions in the CNS. Although IFN-γ has been linked to the death of dopaminergic neurons in PD models (10), contrasting findings indicate that low levels of IFN-γ in a chemically induced model of CNS demyelination exert protective effects on cuprizone-induced gliosis, oligodendrocyte death and the demyelination associated with the up-regulation of insulin-like growth factor-1 (IGF-1) (28, 29). Cross-sectional studies report that, on average, free IGF-1 levels are elevated in patients with T2DM (30). Low levels of IFN-γ in patients with PD–T2DM may exert a protective effect due to the elevation of IGF-1 levels. Interleukin-10, an anti-inflammatory cytokine, appears to be beneficial in PD and T2DM. In our study, a positive correlation was found between IL-10 levels and the severity of depression in patients with PD–T2DM. Although the serum level of IL-10 in patients with PD–T2DM is consistently lower than that in patients with PD, the possibility exists that a higher production of IL-10 in more severe forms of PD with T2DM co-morbidity serves as a compensatory mechanism that attempts to resolve the detrimental inflammatory condition associated with the disease over time.

To the best of our knowledge, this is the first study to investigate the association between peripheral inflammation and motor and non-motor symptoms through the Parkinson’s Disease Scale of Traditional Chinese Medicine and Western Medicine in patients with PD–T2DM. Nevertheless, our study has some limitations. First, the sample size was relatively small, which may have hindered the detection of differences in some inflammatory markers and the evaluation of PD motor and non-motor symptom scales. We corrected for age and gender in statistical analyses, but the age and gender mismatch in the healthy control group may introduce confounding variables. In addition, although the sample size of this study is not based on an efficacy calculation, it provides a preliminary evaluation of the relationship between PD, T2DM and immune markers. Nevertheless, the lack of an *a priori* power calculation may limit the universality of the research results. Second, patients with PD received anti-Parkinsonian medication, and patients with T2DM received antidiabetic medication. However, the effects of dopaminergic and hypoglycaemic drugs on the levels of inflammatory cytokines in patients with PD and T2DM remain unknown. Furthermore, the use of non-steroidal and other drugs may affect levels of inflammatory markers. Patients’ medication histories should be recorded in detail, and the influence of drug use should be adjusted in statistical analyses. Third, there may be selection bias and information deviation in the experimental design. The experiment was based on the actual number of cases recruited, the sample size not having been calculated in advance, and this may affect the statistical significance of the research results and the reliability of its conclusions. Moreover, relying on participants’ memories may lead to memory bias, especially when inquiring about past exposure. Due to the limitations of the study design, it was not possible to verify whether cytokine profiles were correlative or causal in this study.

Consistent with its pilot design and exploratory aims, this study has several limitations. Considering that several differences exist in the correlation of inflammatory cytokines with motor and non-motor symptoms in patients with PD and PD–T2DM, further large-scale studies are required. A longitudinal cohort study was conducted to track the time-varying levels of inflammatory markers and the progress of symptoms in patients with PD and T2DM, helping to determine the causal relationship between inflammation and the disease process. It is also possible to design a randomised controlled trial to evaluate the effects of anti-inflammatory drugs, lifestyle changes and other interventions on inflammatory markers and symptoms of PD and T2DM. Furthermore, the sample size should be increased through multicentre cooperation in the future to improve the representativeness and statistical efficiency of the research and ensure the universality of its results.

## Conclusion

This pilot study provides preliminary evidence suggesting that T2DM can exacerbate the motor and non-motor symptoms of PD and that peripheral inflammatory cytokines can be considered biomarkers in PD–T2DM. The levels of these inflammatory cytokines may provide more information to help predict the progression of the condition in patients with PD–T2DM (namely, changes in motor and non-motor symptoms) and develop reasonable treatment plans. Additionally, the use of appropriate drugs to reduce inflammatory cytokine levels may have a favourable effect on symptoms in patients with PD–T2DM. The underlying mechanism by which T2DM exacerbates the motor and non-motor symptoms of PD may involve increased inflammation.

## Data Availability

The original contributions presented in the study are included in the article/Supplementary material, further inquiries can be directed to the corresponding author.
