# Supplementary material for: Association of peripheral inflammatory cytokines with motor and non-motor symptoms in patients with Parkinson’s disease and type 2 diabetes mellitus
Source: Front Neurol. 2025 Jul 2;16:1474812. doi: 10.3389/fneur.2025.1474812 (PMC12263359; doi:10.3389/fneur.2025.1474812)
Supplement: Supplementary file 1 [file Table_1.DOCX]

The partial correlation between clinical variables and serum inflammatory cytokine levels

|  | TNF-α | IL-1β | IL-6 | IFN‐γ | IL-4 | IL-10 |  |
| --- | --- | --- | --- | --- | --- | --- | --- |
| UPDRS2 | 0.137 | 0.341 | 0.101 | -0.094 | 0.169 | 0.156 | r |
|  | 0.576 | 0.153 | 0.681 | 0.702 | 0.49 | 0.523 | *P* |
| UPDRS3 | 0.267 | -0.225 | -0.497 | 0.355 | -0.095 | -0.181 | r |
|  | 0.269 | 0.355 | 0.031 | 0.135 | 0.699 | 0.459 | *P* |
| Total-UPDRS | 0.449 | 0.088 | -0.256 | 0.205 | -0.246 | 0.044 | r |
|  | 0.054 | 0.719 | 0.290 | 0.401 | 0.311 | 0.858 | *P* |
| NMSS | 0.188 | 0.379 | 0.119 | -0.419 | 0.019 | 0.378 | r |
|  | 0.441 | 0.109 | 0.629 | 0.074 | 0.939 | 0.110 | *P* |
| HAMD | -0.014 | 0.361 | 0.211 | -0.149 | -0.276 | 0.512 | r |
|  | 0.954 | 0.129 | 0.385 | 0.543 | 0.253 | 0.025 | *P* |
| PDSS | -0.287 | 0.266 | 0.212 | 0.095 | 0.062 | 0.330 | r |
|  | 0.233 | 0.271 | 0.383 | 0.698 | 0.800 | 0.168 | *P* |
| SCOPA | -0.138 | 0.205 | 0.281 | -0.588 | 0.222 | 0.151 | r |
|  | 0.573 | 0.401 | 0.244 | 0.008 | 0.360 | 0.538 | *P* |
| PDQ39 | 0.094 | 0.462 | 0.288 | -0.053 | 0.246 | 0.170 | r |
|  | 0.703 | 0.046 | 0.232 | 0.830 | 0.310 | 0.485 | *P* |
| COURSE | 0.689 | -0.102 | -0.219 | 0.083 | -0.265 | -0.013 | r |
|  | 0.001 | 0.679 | 0.369 | 0.735 | 0.272 | 0.959 | *P* |
